# A role for divalent metal transporter (DMT1) in mitochondrial uptake of iron and manganese

**DOI:** 10.1038/s41598-017-18584-4

**Published:** 2018-01-09

**Authors:** Natascha A. Wolff, Michael D. Garrick, Lin Zhao, Laura M. Garrick, Andrew J. Ghio, Frank Thévenod

**Affiliations:** 1 Department of Physiology, Pathophysiology & Toxicology and Center for Biomedical Education and Research (ZBAF), University of Witten/Herdecke, D-58453 Witten, Germany; 2 Department of Biochemistry, State University of New York (SUNY), Buffalo, New York 14214 USA; 3 National Health and Environmental Effects Research Laboratory, Office of Research and Development, U.S. Environmental Protection Agency, Chapel Hill, North Carolina 27599–7315 USA

## Abstract

Much of iron and manganese metabolism occurs in mitochondria. Uptake of redox-active iron must be tightly controlled, but little is known about how metal ions enter mitochondria. Recently, we established that the divalent metal transporter 1 (DMT1) is present in the outer mitochondrial membrane (OMM). Therefore we asked if it mediates Fe^2+^ and Mn^2+^ influx. Mitochondria were isolated from HEK293 cells permanently transfected with inducible rat DMT1 isoform 1 A/+IRE (HEK293-rDMT1). Fe^2+^-induced quenching of the dye PhenGreen™SK (PGSK) occurred in two phases, one of which reflected OMM DMT1 with stronger Fe^2+^ uptake after DMT1 overexpression. DMT1-specific quenching showed an apparent affinity of ~1.5 µM for Fe^2+^and was blocked by the DMT1 inhibitor CISMBI. Fe^2+^ influx reflected an imposed proton gradient, a response that was also observed in purified rat kidney cortex (rKC) mitochondria. Non-heme Fe accumulation assayed by ICPOES and stable ^57^Fe isotope incorporation by ICPMS were increased in HEK293-rDMT1 mitochondria. HEK293-rDMT1 mitochondria displayed higher ^59^Fe^2+^ and ^54^Mn^2+^ uptake relative to controls with ^54^Mn^2+^ uptake blocked by the DMT1 inhibitor XEN602. Such transport was defective in rKC mitochondria with the Belgrade (G185R) mutation. Thus, these results support a role for DMT1 in mitochondrial Fe^2+^ and Mn^2+^ acquisition.

## Introduction

Mitochondria are major sites of iron and manganese utilization. Mn^2+^ plays an important role in antioxidant defence as a cofactor of the matrix enzyme superoxide dismutase 2^1^ whereas Fe^2+^ is incorporated in heme and iron sulphur clusters that are synthesized in the mitochondrial matrix^2,3^. As almost all iron in the circulation is bound to transferrin under physiological conditions, cellular iron acquisition mainly occurs by transferrin receptor-mediated endocytosis. Following reduction by endosomal ferrireductases^4^ and release from transferrin, iron is exported from the endosomal compartment by the divalent metal transporter DMT1 (DCT1, Nramp2, SLC11a2)^5^ that also plays an important role in mediating non-heme iron absorption across the duodenal brush border^6^. Typically, DMT1 operates as a metal-proton cotransporter^7^. The substrate spectrum of DMT1 includes several divalent metal ions with low micromolar affinity, including Fe^2+^, Mn^2+^, Cd^2+^ and Co^2+^, while Zn^2+^ appears to be a poor substrate, and Fe^3+^ is clearly not transported^7–10^.

Cytosolic iron could be bound in low MW complexes or, more likely, to chaperone proteins for trafficking to the sites of cellular storage or use^11,12^. Alternatively, iron may be delivered from endosomes to mitochondria by direct contact between the organelles and subsequent interorganellar transfer, called a “kiss-and-run” mechanism^13^, now supported in both erythroid^13^ and non-erythroid cells^14^.

Whatever the route of delivery, iron has to cross two membranes to enter the mitochondrial matrix, the outer (OMM) and inner mitochondrial membrane (IMM). The OMM is widely believed to be well permeable to small solutes due to the existence of relatively large pores, at least partially represented by voltage-dependent anion channels (VDACs, porins)^15,16^. Nevertheless, Fe^2+^ flux through VDAC has, to our knowledge, never been demonstrated, neither in the poorly cation permeable open state^17^, nor in the closed state favoured by the electrical potential difference across the OMM^17–19^ inferred from the pH measurements of Porcelli and coworkers^20^. Mitoferrins mediate Fe^2+^ flux across the IMM with potential additional pathways playing only a minor role in mammalian cells, at least in mouse 3T3 fibroblasts grown with about 2 µM iron^21^. Little is known, however, about the characteristics of mitoferrin-mediated transport in terms of substrate selectivity, affinity or driving force(s).

Using a variety of methods, we have previously obtained evidence for the expression of DMT1 also in mitochondria in cell lines and tissues from various species^22,23^. Cytochrome C oxidase subunit II, one of two mitochondrial proteins identified in a split-ubiquitin yeast two-hybrid screen for putative DMT1 interaction partners, co-immunoprecipitated with DMT1. Moreover, immunoblots of the OMM fraction isolated from rat kidney cortex displayed substantially increased DMT1 reactivity compared to just isolated mitochondria. Using HEK293 cells that inducibly express either DMT1 1A/ + IRE or DMT1 1B/-IRE, we found both isoforms in the OMM, as detected by immunoblots after cell fractionation, or in isolated mitochondria, as detected by immunofluorescence microscopy. Mitochondrial DMT1 immunoreactivity and co-localization with VDAC was also observed by immunogold labelling in rat renal cortex sections.

Mere localization of DMT1 in mitochondria does not provide evidence for a functional relevance in divalent metal homeostasis of this organelle. The present study assesses the role of DMT1 in mitochondrial metal ion transport, using isolated mitochondria from DMT1-overexpressing HEK293 cells as well as from kidney cortex tissue of normal and the DMT1-deficient Belgrade rats. Multiple approaches enable the advantages of individual methods to compensate for occasional limitations. Methods include uptake and accumulation measurements with labelled and unlabelled divalent metals, and particularly monitoring of metal-induced quenching of the indicator dye Phen Green™ SK (PGSK) preloaded into the mitochondria. The results support the hypothesis that DMT1 is involved in mitochondrial iron and manganese acquisition.

## Materials and Methods

### Materials

Tet system-suited fetal bovine serum (FBS) was obtained from Clontech (Takara Bio Europe, Saint-Germain-en-Laye, France), Fisher Scientific (Schwerte, Germany) or Atlanta Biological (Atlanta, GA), hygromycin from Carl Roth (Karlsruhe, Germany) or Invitrogen (Carlsbad, CA), and Geneticin (G418) from Biochrom (Berlin, Germany) or Invitrogen (Carlsbad, CA). PGSK diacetate (Molecular Probes) was purchased from Fisher Scientific (Schwerte, Germany). Rotenone (Calbiochem) was from Merck Millipore (Darmstadt, Germany), and 2-(3-carbamimidoylsulfanylmethyl-benzyl)-isothiourea (CISMBI), poly-D-Lysine hydrobromide, doxycycline hyclate, sodium succinate, ferrous sulphate, ferric ammonium citrate (FAC), manganese(II) sulphate, iron(III) chloride, and protease inhibitor cocktail were obtained from Sigma-Aldrich (Taufkirchen, Germany or St. Louis, MO). XEN602 and recombinant human erythropoietin were generously provided by Xenon (Burnaby, BC, Canada) and Amgen (Thousand Oaks, CA), respectively. ^59^FeCl_3_ and ^54^MnCl_2_ were from Perkin-Elmer (Waltham, MA). ^57^Fe- FAC was from Cambridge Isotope Laboratories, Inc., Andover, MA

### Culture of HEK293 cells permanently transfected with DMT1

The establishment of HEK293 cells permanently transfected with rat DMT1 isoform 1 A/+IRE under the control of a Tet-On promoter was previously described^24^. Cells were cultured as described before^24^ in poly-D-lysine-treated culture vessels. For mitochondrial preparations, cells were seeded at a density to reach about 90–95% confluence at harvest five days later. Induction of DMT1-expression was at 25 nM doxycycline (Dox) for 48 h or at 50 nM for 24 h before the cells were used^25^, and was verified on both the mRNA level by reverse transcription-polymerase chain reaction and the protein level by immunoblotting.

### Culture of Murine Erythroleukemia (MEL) and CD34 cells

MEL cells (strain BB88) were cultured as described^26^ and induced to differentiate in the presence of 1% (v/v) dimethylsulphoxide (DMSO). Human CD34 cells, purchased from the Fred Hutchinson Cancer Research Centre, were cultured and induced to the designated stages of differentiation according to Lee *et al*.^27^.

### Preparation of mitochondria from rat renal cortex and rDMT1 1A/+IRE-HEK cells

Procedures and animal handling were in accordance with German law on animal experimentation and the European Directive on the Protection of Animals used for Scientific Purposes (2010/63/EU) and approved by the governmental animal ethics committee of North-Rhine-Westphalia, Germany (Landrat Ennepe-Ruhrkreis, Aktenzeichen 32/7 from January 20, 2014; CD rats). Those studies with Belgrade and Long Evans rats were exempt according to the Institutional Animal Care and Use Committee because rats were undergoing a planned cull from a Belgrade rat colony (we thank Dr. Wojciech Krzyzanski and Dr. Monika Krzyzanska for making the culled rats’ kidneys available) and a separate Long Evans rat colony (we thank Dr. Alexis Thompson and Mr. Mauricio Suarez for making the culled rats’ kidneys available). Homozygous (*b/b*) Belgrade rats have a G185R mutation in DMT1 that eliminates most transport activity^5^.

For PGSK experiments, crude mitochondria were obtained as described by Ott *et al*.^28^ with slight modifications: 1) the homogenization buffer contained 2 mM EGTA; 2) the mitochondrial pellet was resuspended in mitochondria resuspension buffer (MRB; in mM: 210 mannitol, 70 sucrose, 3 HEPES/KOH, pH 7.0). Likewise, for rDMT1-HEK293 cells, the protocol was modified as follows: 1) cells were disrupted by nitrogen cavitation at 300 p.s.i. (pounds per square inch), for 2 min); after low speed centrifugation, mitochondria in the supernatant were separated by centrifugation (9,000 × g, 10 min) and resuspended in MRB. These procedures were carried out at 4 °C.

For the ICPOES and ICPMS experiments, mitochondria were isolated by differential centrifugation according to Greenawalt^29^. To assay for incorporation of radioactively labelled metal ions or do immunoblotting, mitochondria were prepared using Thermo (Rockford, IL) kits for isolation from cells (cat. #89874) or renal cortical tissue (cat. #89801). Where the kits involved a choice for procedure, the choice was always to maximize purity rather than recovery.

### Metal transport assays

#### ICPOES and ICPMS

To assay for accumulation of non-heme iron from non-labelled FAC, we exposed mitochondria to either 1) media or 2) media with 100 µM FAC for 4 h. After centrifugation (20,000 g × 10 minutes), the mitochondria were washed with PBS and centrifuged again. After hydrolysis in 1.0 mL 3 N HCl/10% trichloroacetic acid for 24 h, the non-heme iron concentration of the mitochondria was measured using inductively coupled plasma optical emission spectroscopy (ICPOES; Model Optima 4300D, Perkin Elmer, Norwalk, CT) operated at a wavelength of 238.204 nm.

In another series of experiments, mitochondria were exposed to either 1) media or 2) media with 10 µM ^57^Fe FAC for 4 h. After centrifugation (20,000 g × 10 minutes), the mitochondria were gently washed with PBS and centrifuged again. The mitochondria were hydrolysed in 1.0 mL 3 N HCl/10% trichloroacetic acid for 24 h. The concentration of ^57^Fe was measured by ICP mass spectroscopy (ICPMS; Elan DRC II, Perkin Elmer).

#### Radioisotope tracers

Mitochondria were incubated as described previously^24^. ^59^FeCl_3_ was mixed with a 10x excess of FeSO_4_ then a 100x excess of ascorbate to give a final [Fe^2+^] = 2μEq/L, or ^54^MnCl_2_ was mixed with a 10x excess of MnSO_4_ to give a final [Mn^2+^] = 0.5μEq/L. After the incubations, processing mitochondria and counting incorporation was also done as described before^24^.

#### PGSK Fluorescence

For experiments with the membrane-permeant metal ion indicator PGSK diacetate, mitochondria were preloaded with 50 µM of the dye for 1 h at 21 °C in an orbital shaker essentially as described by Chavez-Crooker *et al*.^30^, except that rotation was reduced to 25 revolutions min^−1^. Mitochondria were pelleted (2000xg, 10 min), washed once with MRB, centrifuged as before, resuspended in MRB and maintained on ice. PGSK fluorescence was monitored at 21 °C in a Perkin Elmer LS50B luminescence spectrometer at 506 nm λ_ex_ and 532 nm λ_em_, slit widths 10 nm, and with a lower emission cut-off of 515 nm. Mitochondria (<3 vol. %) were suspended in 2 ml assay buffer (210 mannitol, 70 sucrose, 3 HEPES/KOH, and adjusted to the pH-value indicated). Mitochondria were equilibrated with the assay buffer for 10 min before starting fluorescence measurements, unless acute pH effects were investigated, where mitochondria were added immediately after starting the recording. For experiments with the DMT1 inhibitor CISMBI, mitochondria were pre-incubated with the compound for 10 min before addition of Fe^2+^. For determination of Fe^2+^ affinity, experiments were performed at pH 7.6. Stocks of freshly prepared FeSO_4_ containing 125 mM ascorbate were added at the concentrations indicated (250 µM final concentration of ascorbate in assay). The decrease in fluorescence (ΔF), indicating metal-dependent quenching and thus metal uptake by mitochondria, was normalized to the starting point of the second phase and measured for 20 sec, with the exception of the CISMBI experiments where effects of the compound were monitored for a period of 450 sec to account for a slow onset of inhibition.

### Preparation of cytosol from HEK293 cells

Confluent cells from 6 × 175 cm^2^ growth area were suspended in 1 ml MSH buffer including 1/6 vol. protease inhibitor cocktail (Sigma-Aldrich) and disrupted by nitrogen cavitation as described above. The membranous material was cleared by centrifugation at 150,000 x g for 1 h.

### Immunoblotting and protein determination

Electrophoresis, immunoblotting and protein determination were done as before using two anti-DMT1 antibodies directed against peptide sequences in exon 2 (ex2) or the 4^th^ extracellular region (4ec)^22^.

### Statistics

Unless otherwise indicated, data are means ± SEM of the number of independent experiments given in the figure legends. For more than two groups, one-way ANOVA with Bonferroni post-hoc test was performed using GraphPad Prism version 5.01 (La Jolla, CA). Results with P ≤ 0.05 were considered to be statistically significant. Kinetic data of Fe^2+^-induced PGSK quenching were fitted to a one-site saturation binding function $\% {\rm{\Delta }}F=\frac{ \% {\rm{\Delta }}{F}_{\max }\times [F{e}^{2+}]}{{K}_{0.5}^{F{e}^{2+}}+[F{e}^{2+}]}$ also using SigmaPlot ver. 12.5. For comparisons between two groups, unpaired two-tailed Student’s t-test was performed with SigmaPlot ver. 12.5 (Systat Software, Erkrath, Germany). Data in radioisotope incorporation experiments were fitted by regression and analysed by ANOVA using Stata ver. 13.1 (Stata Corp., College Station, TX).

### Data availability

The datasets generated during and/or analysed during the current study are available from the corresponding authors on reasonable request.

## Results

### rDMT1 overexpression increases mitochondrial Fe^2+^ uptake measured with PGSK

**Figure 1:**
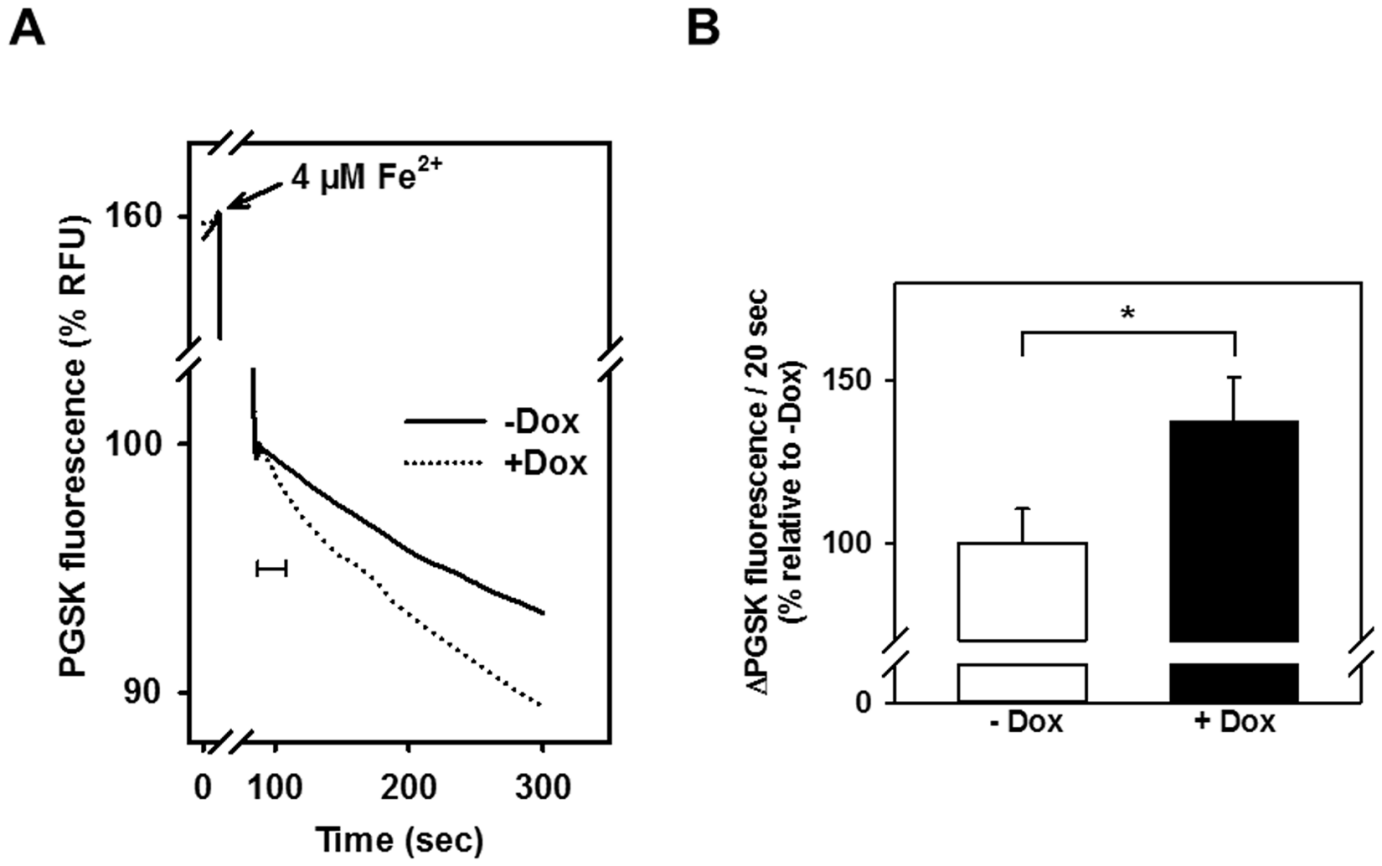
Mitochondrial Fe^2+^ uptake is increased after doxycycline induction of rDMT1 in HEK293 cells. Mitochondria were isolated from uninduced (−Dox.) or 48 h with 25 nM doxycycline induced ( + Dox.) rDMT1-HEK293 cells, loaded with PGSK, and subsequently washed as indicated in Materials and Methods. After 10 min of pre-incubation at pH 7.0, PGSK fluorescence was measured at λ_ex_ = 506 nm and λ_em_ = 532 nm. (**A**) Representative traces of the time course of the fluorescence after the addition of 4 µM Fe^2+^. Curves are normalized to fluorescence at the beginning of the second phase (100%). (**B**) Statistical analysis of second phase PGSK quenching. Initial slopes of quenching, as indicated by the 20s-scale bar in A, were normalized to measurements in mitochondria isolated from uninduced cells. Means ± SEM of 26 measurements in 10 independent experiments are shown. *P < 0.05. RFU, relative fluorescence units.

Our previous studies showed the localization of DMT1 in the OMM^22,23^. Therefore we asked if Fe^2+^ uptake by mitochondria isolated from HEK293 cells stably transfected with rat 1 A/ + IRE DMT1 reflected the Tet-on control of the CMV promoter (Fig. 1). Fe^2+^ uptake by mitochondria isolated from uninduced and doxycycline-induced HEK293 cells was assayed using the fluorescent metal ion indicator PGSK; PGSK is quenched by chelatable iron and other metal ions, making it useful to test metal ion transport across biological membranes^30,31^. PGSK quenching followed a biphasic time course after addition of 4 µM Fe^2+^, with an initial fast phase, followed by a slow second phase (Fig. 1A). When mitochondria from cells without and with Dox induction were compared, only the second phase was significantly increased by DMT1 overexpression (Fig. 1B), suggesting that the 2^nd^ phase represents mitochondrial Fe^2+^ uptake mediated by DMT1. Notably, when Fe^3+^ was added at matching concentration, PGSK fluorescence did not show sustained quenching as observed for Fe^2+^, but rapidly returned to the initial value after mixing (data not shown), although PGSK is sensitive to Fe^3+^ at low micromolar concentrations^32^.

### Properties of the second Fe^2+^ uptake phase reflect those of DMT1

**Figure 2:**
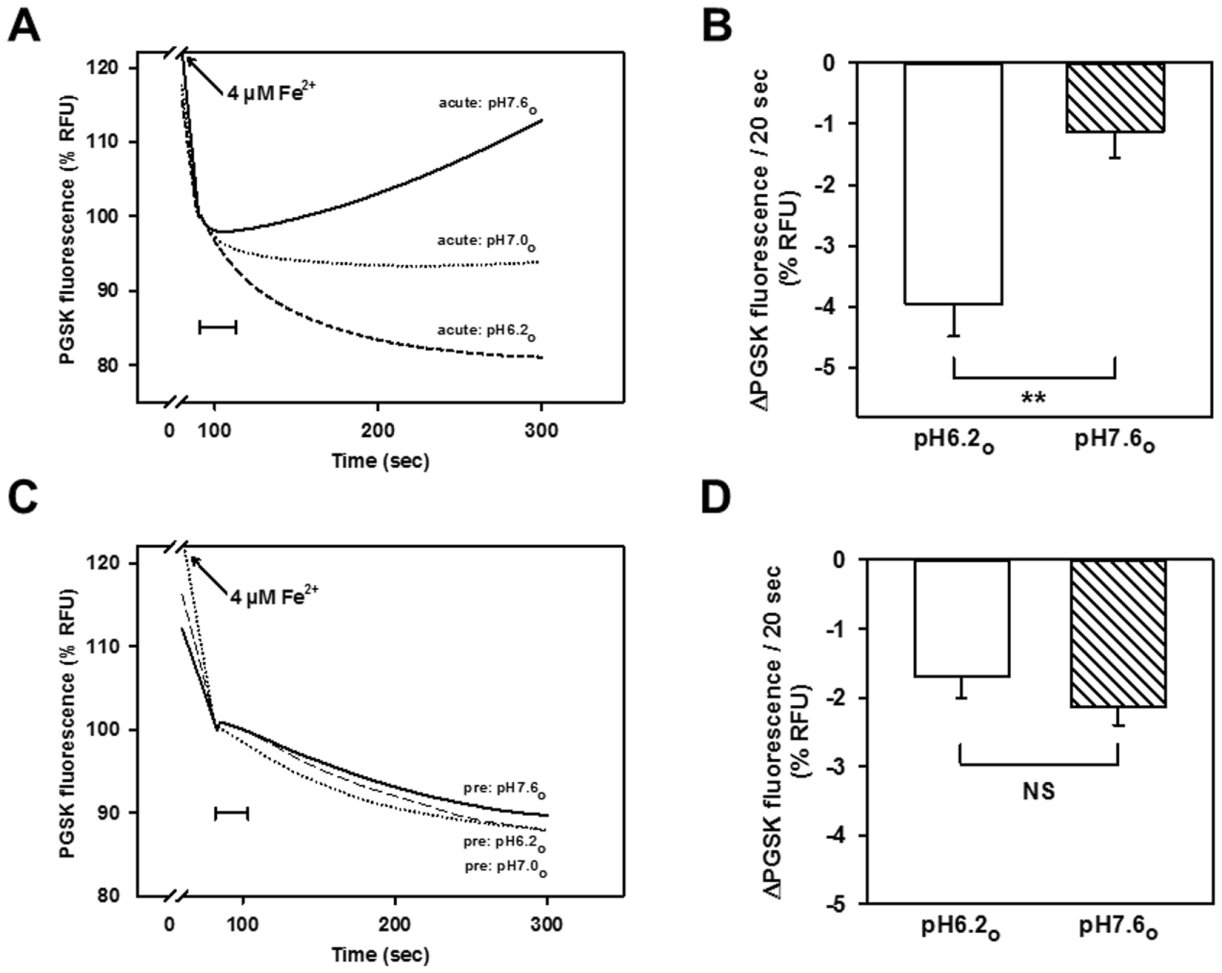
Fe^2+^ uptake in mitochondria overexpressing rDMT1 is increased by an inward-directed proton gradient. PGSK-loaded mitochondria (pH 7.0_i_) from doxycycline-induced rDMT1-HEK293 cells were suspended in pH 6.2_o_, 7.0_o_, or 7.6_o_ assay buffer immediately (**A**,**B**) or pre-equilibrated for 10 min in the assay buffer (**C**,**D**) prior to quenching induced by 4 µM Fe^2+^. (**A**,**C**) Representative traces of the fluorescence time course are shown. (**B**,**D**) Statistical analyses of second phase PGSK quenching. Initial slopes of quenching, as indicated by the 20s-scale bars in (**A** and **C**), are plotted as means ± SEM (8–9 measurements in 4 independent experiments) for pH6.2 and pH7.6, respectively. Fluorescence quenching (−ΔPGSK), reflecting Fe^2+^ uptake, was normalized to the respective initial value of the second phase that was set to 100%. **P < 0.01; NS, not significant. RFU, relative fluorescence units.

Since DMT1 typically operates as a metal-H^+^ cotransporter^7^, H^+^-driven mitochondrial Fe^2+^ uptake was tested in rDMT1-overexpressing HEK293 cells by rapidly suspending mitochondria (pH 7.0) in buffers at different pH values (6.2, 7.0 or 7.6) and immediately adding Fe^2+^, or by pre-equilibrating mitochondria for 10 min in the buffers prior to addition of Fe^2+^. As shown in Fig. 2A, second-phase Fe^2+^-induced PGSK quenching was more pronounced in the presence of an acute inwardly directed H^+^ gradient (*pH6*.2_*o*_ *versus 7*.0_*i*_) compared to the condition where mitochondria had been pre-equilibrated with the medium (*pH*_*i*_ = *pH*_*o*_), whereas an opposite proton gradient (*pH7*.6_*o*_ *versus 7*.0_*i*_) not only abrogated the secondary fluorescence decrease, but also resulted in a gradual signal recovery, suggesting Fe^2+^ efflux. Quantification of the initial (20 sec) slopes of the second-phase fluorescence decline yielded a significant difference between pH7.6 and pH6.2 (Fig. 2B). In contrast, when the same external pH values were applied under equilibrated conditions, there was no difference in second-phase uptake between pH 7.6, 7.0 and 6.2 (Fig. 2C), with no significant effect of pH on its initial slope (Fig. 2D), which indicates that the proton gradient, rather than the proton concentration per se (i.e. a pH effect), enhances second-phase influx at pH6.2 compared to pH7.6. These findings indicate the contribution of a proton-driven pathway to second-phase Fe^2+^ uptake, consistent with the involvement of DMT1.

**Figure 3:**
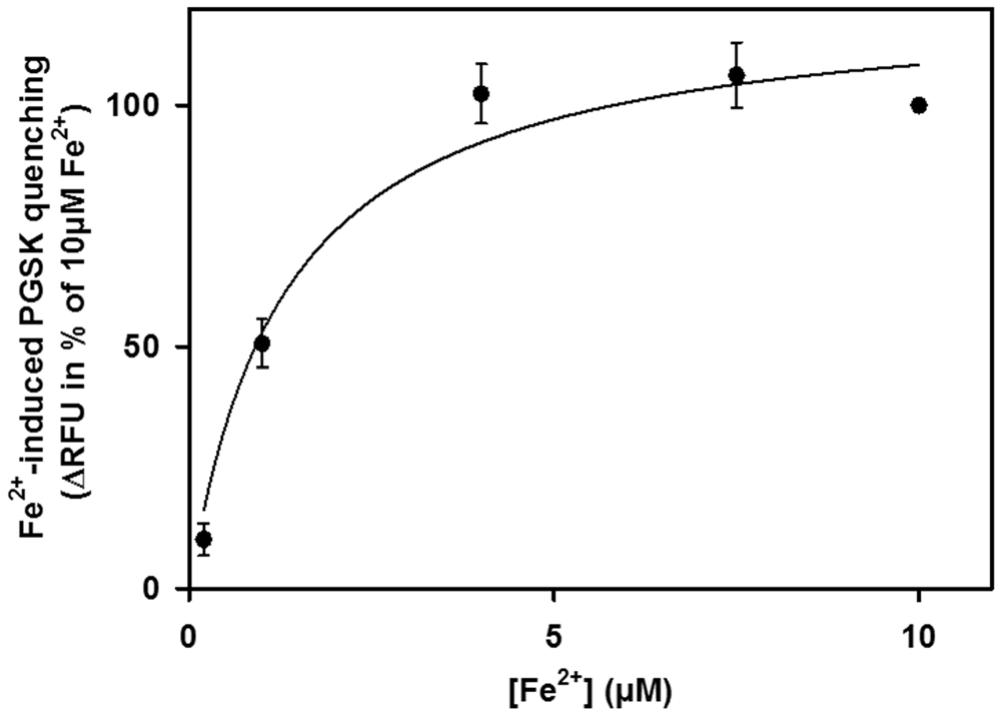
Apparent affinity of second phase Fe^2+^ uptake in mitochondria from rDMT1-overexpressing HEK293 cells is consistent with DMT1-mediated transport. PGSK-loaded mitochondria (pH 7.6_i_) from doxycycline-induced rDMT1-HEK293 cells were assayed for quenching induced by increasing Fe^2+^ concentrations. Fe^2+^ was added from 500x concentrated FeSO_4_ stock solutions, which had been supplemented with 50% cytosol, as detailed in Materials and Methods. The initial 20s-slopes of second-phase PGSK quenching (−ΔPGSK), as described in Fig. 1, were normalized to values obtained at the maximally tested concentration of 10 µM Fe^2+^. Data represent means ± SEM of 4–7 independent experiments. Data were fitted to a one-site saturation binding function, yielding a half-saturation concentration $({K}_{0.5}^{F{e}^{2+}})$ of 1.3 µM Fe^2+^. RFU, relative fluorescence units.

DMT1 has been reported to have an affinity for Fe^2+^ in the 1–2 µM range, or even below 1 µM^7,24,33^. We therefore assayed for concentration dependence of Fe^2+^-induced PGSK quenching in mitochondria from rDMT1-overexpressing HEK293 cells. As Fe^2+^ is unlikely ever to be free in the cytosol, but rather bound in low-molecular weight complexes or to cytosolic chaperones (reviewed in^34^), Fe^2+^ solutions for these experiments were supplemented with HEK293 cytosol (to 5 mg/ml protein) to approximate physiological conditions more closely. As shown in Fig. 3, the slow second phase of PGSK quenching was almost saturated at about 7.5 µM Fe^2+^, with half-maximal effect at 1.3 µM Fe^2+^, after fitting the data to a one-site saturation binding function (R^2^ = 0.97). Thus, the affinity of the second phase of PGSK quenching by Fe^2+^ is consistent with DMT1-mediated flux, while the first phase showed a 10-fold lower Fe^2+^ affinity (Supplemental Fig. S1).

**Figure 4:**
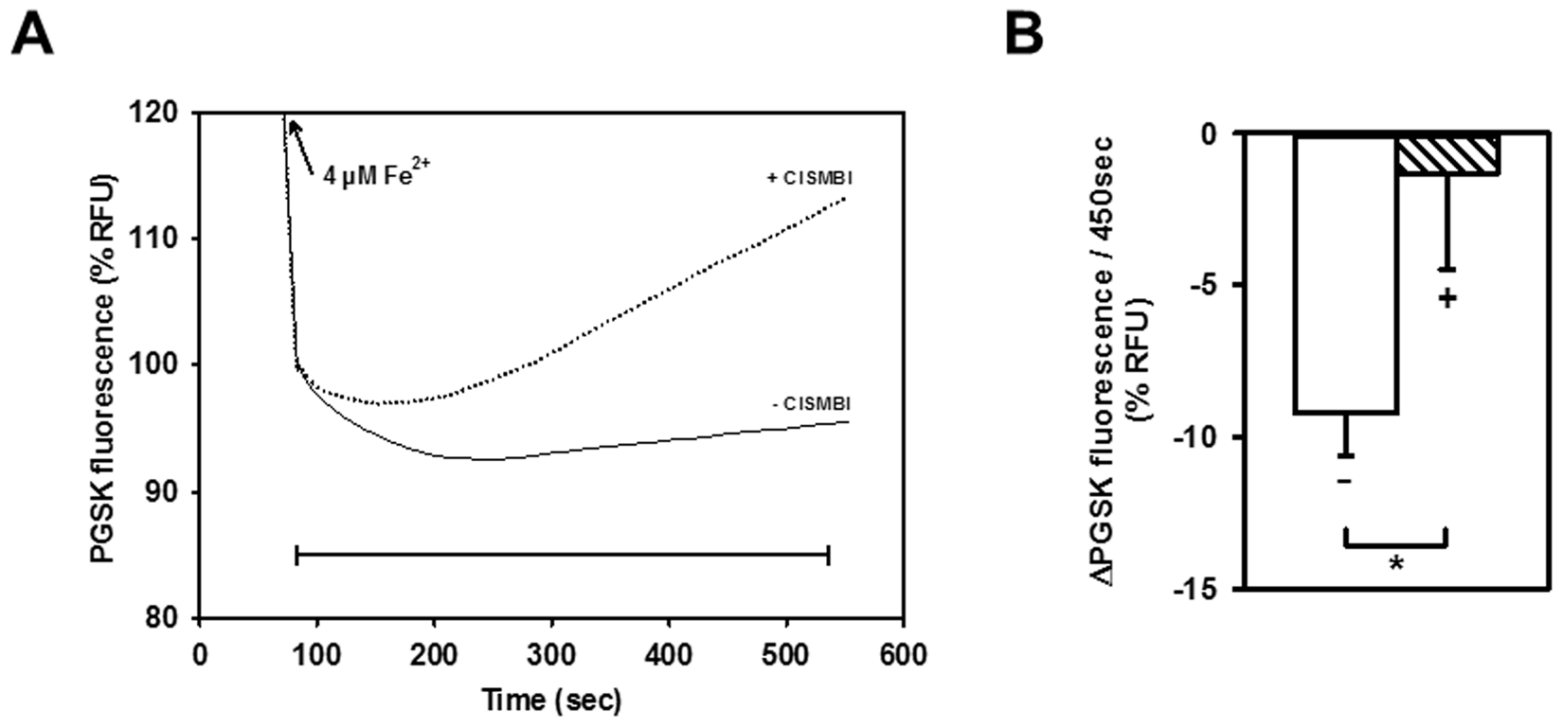
Fe^2+^ uptake in mitochondria from rDMT1-overexpressing HEK293 cells is decreased by the DMT1 inhibitor CISMBI. PGSK-loaded mitochondria (pH 7.0_i_) from doxycycline-induced rDMT1-HEK293 cells were assayed for quenching induced by 4 µM Fe^2+^ in the absence or presence of 0.1 mM of the competitive DMT1 inhibitor CISMBI that had been pre-incubated for 10 min prior to addition of Fe^2+^. (**A**) Representative kinetics of fluorescence changes after addition of 4 µM Fe^2+^ are normalized to the initial fluorescence of the second phase (100%). (**B**) Statistical analysis of second phase PGSK quenching. Fluorescence quenching (−ΔPGSK) over 450 sec (to account for a slow onset of inhibition), as indicated by the scale bar in A, is plotted as means ± SEM of 12 measurements in 4 independent experiments. **−**, no CISMBI; + , CISMBI. *P < 0.05. RFU, relative fluorescence units.

2-(3-carbamimidoylsulfanylmethyl-benzyl)-isothiourea (CISMBI) is a competitive inhibitor of DMT1^35^. The effect of CISMBI was tested on Fe^2+^ flux into mitochondria from both Dox-induced and uninduced rDMT1-HEK293 cells. 100 µM CISMBI, a concentration that completely inhibits 5 µM Cd^2+^ uptake into hDMT1-overexpressing HEK293 cells^35^ and that did not itself alter the response of PGSK, dramatically changed the time course of Fe^2+^-induced PGSK quenching in mitochondria from rDMT1-overexpressing cells. In contrast to the gradual increase in quenching observed during the 2^nd^ phase in control mitochondria without inhibitor, with CISMBI the fluorescence change was initially unaffected but then reversed direction, suggesting Fe^2+^ efflux via a CISMBI insensitive pathway (Fig. 4A). The effect of CISMBI on Fe^2+^ uptake over a monitored period of 450 sec was reproducible (Fig. 4B). In contrast, neither the 1^st^ phase nor 2^nd^-phase PGSK quenching for mitochondria from uninduced cells was affected by CISMBI (data not shown).

### rDMT1 overexpression increases mitochondrial Fe^2+^ uptake measured with ICPOES and ICPMS

**Figure 5:**
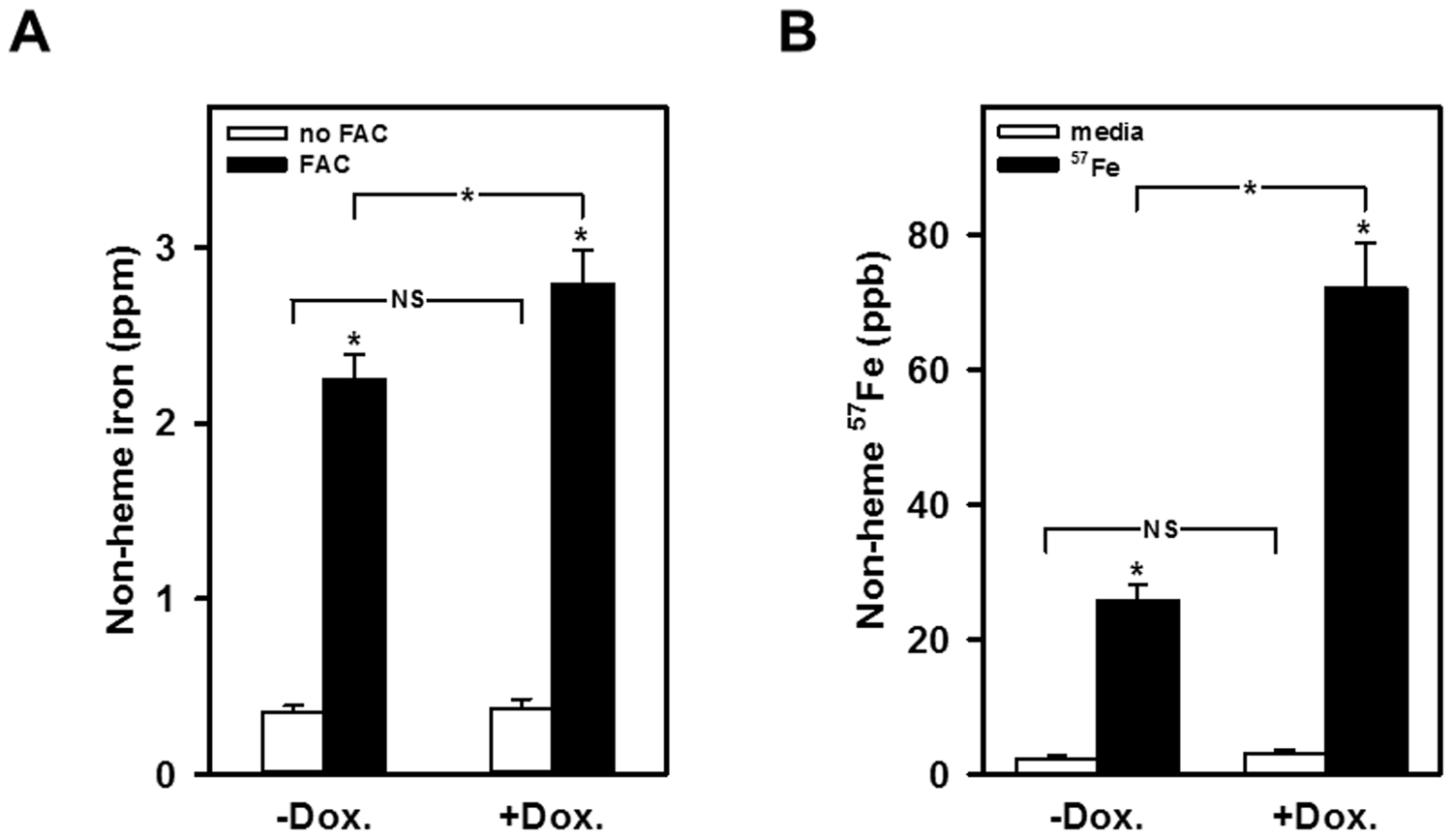
Iron accumulation is increased in mitochondria from rDMT1-overexpressing HEK293 cells, as measured by ICPOES and ICPMS. (**A**) Non-heme iron accumulation. Mitochondria were exposed to medium lacking or containing 100 µM FAC for 4 h. Accumulation of non-heme iron was determined by ICPOES as described in the Methods. (**B)** ^57^Fe incorporation. Mitochondria were exposed to medium lacking or containing 10 µM ^57^Fe-FAC for 4 h. Incorporation of non-heme iron was determined by ICPMS as described in the Methods. Data are means ± SD of 6 determinations in two independent experiments, respectively. *Denotes statistical significance of differences on the *P* < 0.01 level as calculated using one-way analysis of variance. NS, not significant.

Because PGSK fluorescence quenching is an indirect measure of transport, we also tested the dependence of mitochondrial iron accumulation on DMT1 by two independent techniques that rely on much longer incubations: uptake was first measured by ICPOES which demonstrated that induced mitochondrial DMT1 expression increased the non-heme iron level to a value significantly above the uninduced level (Fig. 5A). Similarly, when stable isotope incorporation was assayed by ICPMS, mitochondria again exhibited enhanced levels upon induction of DMT1 expression with doxycycline (Fig. 5B). The 4 h incubation for both parts of Fig. 5 obscured the distinction between phases 1 and 2; instead it makes the difference between induction and its absence less striking but no less reproducible.

### rDMT1 overexpression increases mitochondrial Mn^2+^ and Fe^2+^ uptake by radioisotope measurements and uptake shares properties of DMT1

**Figure 6:**
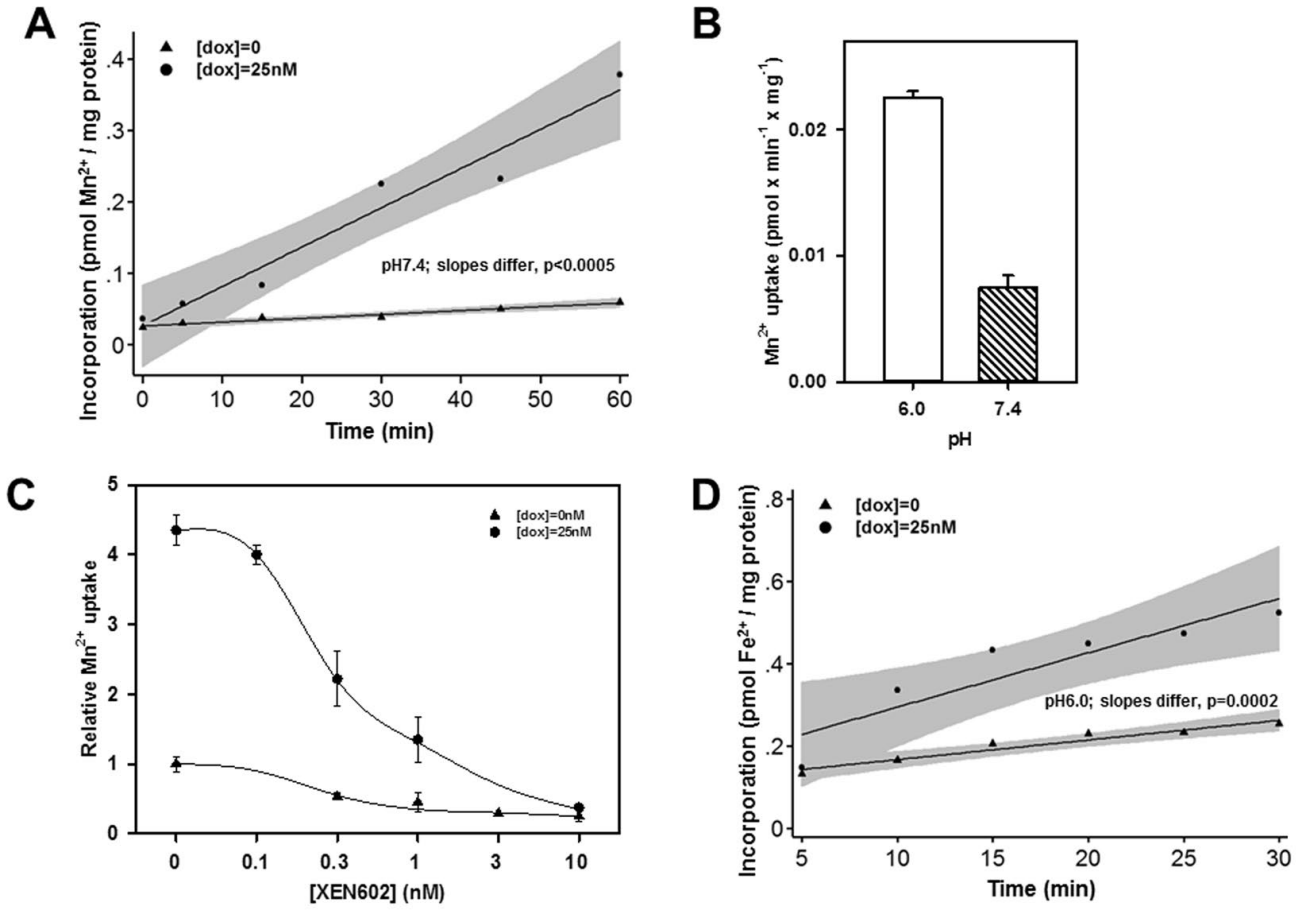
Mitochondrial Mn^2+^ and Fe^2+^ uptake in rDMT1-overexpressing HEK293 cells is consistent with DMT1.Mitochondria were isolated from uninduced ([dox] = 0) or induced ([dox] = 25 nM) rDMT1-HEK293 cells for all 4 panels. (**A)** Time course for Mn^2+^ uptake. Mitochondria were exposed to 0.5 µM ^54^Mn^2+^ at pH 7.4 and incorporation determined as described in the Methods. Lines were fitted by regression; shading indicates 95% confidence intervals. This plot is representative of 3 separate experiments after which all remaining ^54^Mn^2+^ experiments were at pH 6.0. (**B**) pH dependence of Mn^2+^ uptake. Mitochondria were exposed to 0.5 µM ^54^Mn^2+^ at pH 6.0 and 7.4 then incorporation was determined like in (**A**). Only mitochondria from cells exposed to 25 nM doxycycline were analysed. The slope for each plot and the standard error of the estimate (SEE) for the slope were calculated as in (**A**) then plotted as Mn^2+^ uptake and error bars for these values. This plot is representative of 2 separate experiments. (**C**) Inhibition of mitochondrial Mn^2+^ incorporation by XEN602, a specific DMT1 inhibitor. Mitochondria were exposed to 0.5 µM ^54^Mn^2+^ at pH 6.0 and incorporation determined like in (**B**) except that incubations included XEN602 concentrations as indicated. The slope for each plot and the SEE for the slope were calculated as in (**A**) then plotted here as relative Mn^2+^ uptake with uninduced and uninhibited uptake (1.8 pEq Mn × min^−1^ × mg^−1^ protein) set as 1.0 and error bars for these values. This plot is representative of 2 separate experiments. (**D**) Time course for Fe^2+^ uptake. Mitochondria were exposed to 2 µM ^59^Fe^2+^ at pH 6.0 and incorporation was determined as described in the Methods. Lines were fitted by regression; shading indicates 95% confidence intervals. This plot is representative of 6 separate experiments.

Incorporation of ^54^Mn^2+^ or ^59^Fe^2+^ by isolated mitochondria allowed study of intervals shorter than those used by ICP techniques above but longer than when looking at quenching of PGSK fluorescence. When ^54^Mn^2+^ was the DMT1 substrate for uptake by mitochondrial preparations (Fig. 6A), transport rates were enhanced upon induction of DMT1 expression, but the intercepts for Mn incorporation were lower with the initial experiments at pH 7.4. As mentioned above, DMT1 is proton-driven, reflected in an optimum pH of ~6.0 for metal ion uptake^24,36^. Figure 6B demonstrates that ^54^Mn^2+^ uptake was ~3x higher at pH 6 than at 7.4 so pH 6 was used for subsequent experiments. While the Tet-on response provides evidence that the increased uptake is attributable to DMT1 for both Fe and Mn, a specific inhibitor of DMT1 could also test the issue. XEN602 (initially designated XEN601) is such an inhibitor^37^. Mn incorporation is strongly inhibited by XEN602 in induced HEK293 cells (Fig. 6C); 50% inhibition occurs at [XEN602] = ~0.3 nM. Inhibition is also observed in mitochondria from uninduced HEK293 cells, yet the level of isotope incorporation is much smaller than in mitochondria from induced cells and close to background incorporation (Fig. 6C). When transport was alternatively assayed using ^59^Fe^2+^, the rate of uptake again clearly reflected induction of DMT1 by doxycycline, although the intercepts for both lines were well above 0 when the data were extrapolated to 0 time for both uninduced and induced levels of DMT1 (Fig. 6D). Here as in Fig. 5, the time scale for the assay influences what one can see; phase 1 likely contributes to the higher intercept yet there is still a modest but reproducible difference in slopes.

### Mitochondria isolated from rat kidney cortex show Fe^2+^ and Mn^2+^ uptake that is consistent with an involvement of DMT1

**Figure 7:**
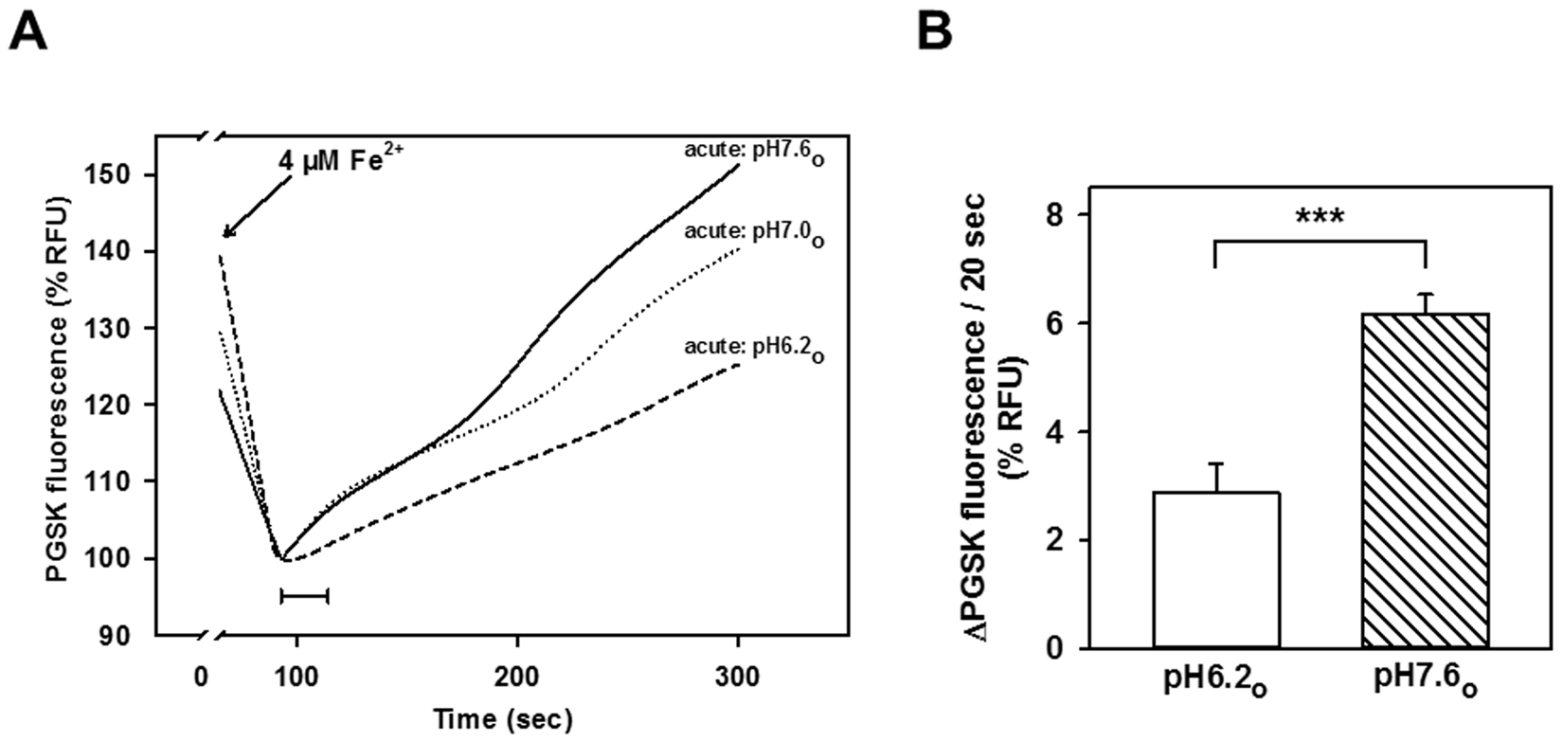
Fe^2+^ efflux from rat kidney cortex mitochondria is strongly reduced by an inward-directed proton gradient. PGSK-loaded mitochondria (pH 7.0_i_) from rKC were suspended in pH 6.2_o_, 7.0_o_, or 7.6_o_ assay buffers and immediately assayed for quenching induced by 4 µM Fe^2+^. (**A**) Representative traces of the fluorescence time course are shown. Fluorescence data were normalized to the respective initial value of the second phase. (**B)** Statistical analyses of second phase PGSK quenching. Initial slopes of quenching, as indicated by the 20s-scale bar in (**A**), are plotted for pH6.2 and pH7.6, and represent means ± SE of 10–11 measurements in 3 independent experiments. ***P < 0.001. RFU, relative fluorescence units.

To assay for the contribution of DMT1 to mitochondrial metal acquisition in a more physiological system, we also assayed for Fe^2+^-induced PGSK quenching in mitochondria isolated from rat kidney cortex (rKC). In contrast to mitochondria from HEK293 cells, rKC mitochondria exhibited pronounced dequenching during the second phase, indicative of effective efflux mechanism/s. Consistent with an opposing proton-driven uptake pathway, however, efflux was substantially reduced under conditions of an inward-directed proton gradient (Fig. 7A,B).

### Defective Mn^2+^ upake in mitochondria isolated from Belgrade rat kidney cortex

**Figure 8:**
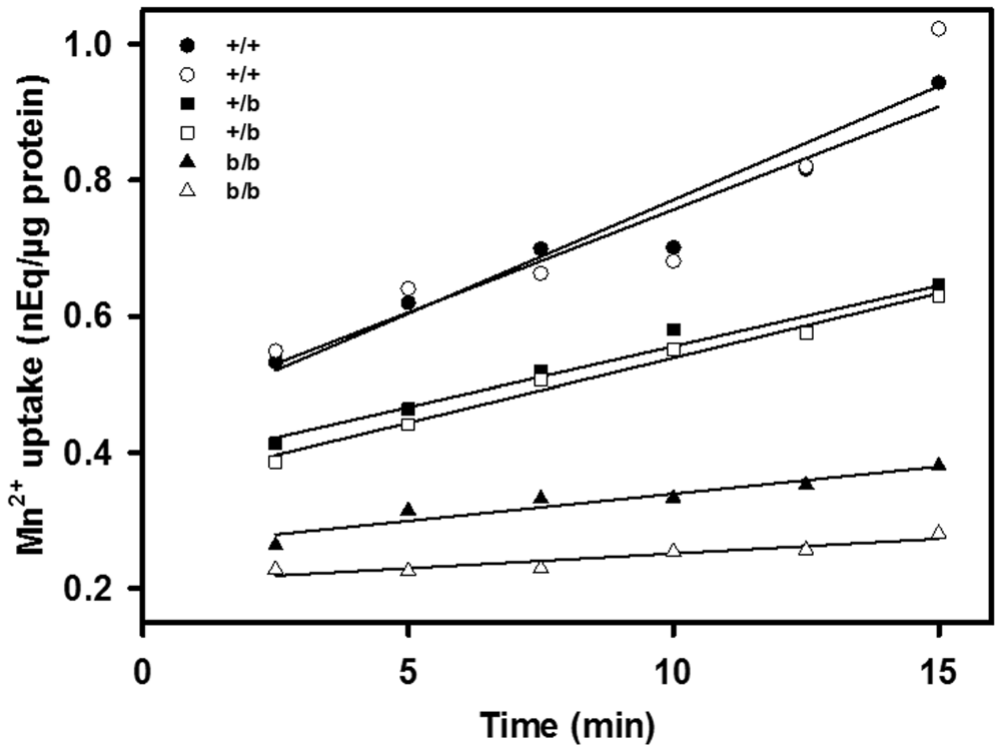
Mn uptake in rat kidney cortex mitochondria depends on the DMT1 genotype.Mitochondria were isolated from two rats of each of 3 genotypes (*b/b* indicating homozygous Belgrade rats with a G185R mutation, +/*b* indicating heterozygous rats and +/+ indicating wild-type animals) then incubated with ^54^Mn^2+^ for the times indicated and incorporation was determined. ANOVA for incorporation based on time, genotype or time/genotype interaction indicated differences for each comparison, P < 0.00005 in all cases as well as for all pairwise genotype comparisons. Designating the mean+/+ slope = 1.0, then one +/+ rat’s slope = 1.05; the other +/+ rat’s slope = 0.95; one +/*b* rat’s slope = 0.56; the other +/*b* rat’s slope = 0.60; one *b*/*b* rat’s slope = 0.25 and the other *b*/*b* rat’s slope = 0.14.

Another way to address whether metal ion uptake by mitochondria exhibits dependency on DMT1 is to learn how mitochondria from animals with mutant DMT1 compare with those from wild-type animals with regard to divalent metal uptake. The Belgrade rat has a G185R mutation in DMT1^5^ (actually G216R in rat 1 A DMT1 due to the 31 amino acid N-terminal extension) that leads to an iron deficient, anemic phenotype due to severely diminished DMT1 transport activity. Mn incorporation by rKC mitochondria reflects the rats’ genotype with wild-type (+/+) rats having the highest rates, homozygous Belgrade (*b/b*) rats having the lowest and heterozygous (+/*b*) rats exhibiting intermediate ones (Fig. 8).

### Cells other than rKC that express high levels of DMT1 in mitochondria

Dox-induced rDMT1-HEK293 cells express high levels of DMT1 in mitochondria but they are engineered to do so. It is not surprising that rKC do too because renal tissue has high DMT1 mRNA levels^38^; this property has served us well in this study and before^22,23^. Future studies need to have more than one tissue where mitochondrial DMT1 plays a role in metal ion metabolism. Erythroid development is a situation where one expects mitochondria in the proliferating stem cell to require only low levels of entry of iron and other metals while commitment to differentiation should lead to much higher OMM DMT1 to address the demand for a 2 × 10^4^-fold accumulation of heme in the erythroid mitochondrion where the last step in heme synthesis occurs^39^. In later stages OMM DMT1 might decline after the demand is being met.

These predictions meet two tests in Fig. S2. Murine Erythroleukemia (MEL) cells proliferate indefinitely under normal culture conditions, but differentiate like erythroid cells when exposed to DMSO^26^. Exposure to DMSO for 1 and 3 days (Fig S2A), leads to increasing hemoglobinization, but mitochondrial DMT1 levels peak at day 1; the 20 kDa subunit of translocase of the outer mitochondrial membrane (TOMM20) serves as a marker for loading of mitochondrial proteins. Human CD34 cells, isolated from bone marrow, are representative of normal hematopoietic stem cells (Fig S2B); they can proliferate extensively but can be induced to go down multiple hematopoietic pathways, particularly the erythroid pathway for which 3 stages are defined^27^. Mitochondrial DMT1 exhibits similar results to those for MEL cells with high levels expressed well before full hemoglobinization develops; cytochrome c (CYT-C) serves as a marker for loading of mitochondrial proteins. The much higher ratio of peak levels of DMT1 to the mitochondrial marker in CD34 cells likely reflects its virtually undetectable concentration at stage 0. Clearly OMM DMT1 is at very high levels in erythroid precursors just when such high levels could support heme synthesis.

## Discussion

Prior evidence for the localization of DMT1 in the OMM^22,23^ suggested its involvement in mitochondrial iron acquisition. To assay for DMT1-dependent transport in mitochondria, in the present study we assessed the uptake of Fe^2+^ and Mn^2+^ by mitochondria isolated from stably transfected HEK293 cells without or with Dox-induced overexpression of rat DMT11A/ + IRE, as well as from rKC. To that end, we used three independent techniques, namely 1) radioisotope uptake, 2) evaluation of iron accumulation by ICPOES and stable isotope-labelled metal ion incorporation measured by ICP mass spectroscopy, as well as 3) measurement of metal-induced quenching of the indicator dye PGSK. PGSK is membrane permeable as a diacetate, but is retained in mitochondria upon cleavage by mitochondrial esterases^31^. This approach offers the advantage of an assessment of the net movement into mitochondria and allows for monitoring of flux kinetics^30^. Tracer assays allow for specific flux measurements of individual metal ions making it an advantage to use multiple approaches. Finally, it is untenable that we were seeing influx into contaminating vesicles because that argument would require that endosomal contaminants would also turn inside out and that the mitochondria, isolated by 3 independent means for which we had initially shown absence of contamination by other cellular fractions^22,23^ were contaminated in all 3 cases.

### Two pathways mediate mitochondrial Fe^2+^ uptake; one is due to DMT1

Using all these approaches to assay divalent metal ion uptake into isolated mitochondria, we observed increased Fe^2+^ and Mn^2+^ uptake in mitochondria from rDMT1 overexpressing HEK293 cells: The second phase of ferrous iron-induced PGSK quenching was significantly enhanced by a prior 48h-treatment of the rDMT1-HEK293 cells with Dox (Fig. 1) to induce OMM DMT1^22,23^, whereas the first phase (see below) was not DMT1 dependent. Interestingly, no quenching response was elicited by ferric iron at the same concentration (data not shown). In agreement with the results obtained with PGSK, ferrous ^59^Fe uptake by rDMT1-HEK293 mitochondria was dependent on induction of DMT1 expression and time (Fig. 6D), but the intercept at t = 0 was considerably > 0. This high intercept had little dependence on DMT1 expression but insight into it came from similar assays (Fig. 6A) of Mn^2+^ uptake that were also dependent on induction of DMT1 expression and time but exhibited a much lower relative level at t = 0. This distinction suggested the existence of two mitochondrial metal ion influx systems with the DMT1-dependent one contributing substantially over time and another one that contributed substantially to Fe^2+^ uptake initially but much less to early Mn^2+^ incorporation. These results demonstrating the dependence on DMT1 for iron accumulation were largely confirmed by ICPOES where induction of mitochondrial DMT1 expression increased non-heme iron level (Fig. 5A) as well as by stable isotope incorporation using ICPMS (Fig. 5B).

Fe^2+^-induced PGSK quenching was strongly stimulated by an acute inwardly directed proton gradient (Fig. 2A,B) relative to pH equilibrium conditions, consistent with DMT1 operating as a proton-metal cotransporter^7^. Only small differences in Fe^2+^-induced PGSK quenching between different pH values were observed under equilibrated conditions (Fig. 2C,D), substantiating the role of the proton gradient in driving Fe^2+^ uptake by DMT1-overexpressing HEK293 mitochondria. Thus, possible non-specific effects of the pH per se on Fe^2+^-induced PGSK quenching would be expected to be apparent under these conditions. In the absence of a proton gradient (pH7.4_i=o_), rat DMT1 has previously been shown to be able to mediate significant metal ion uptake^24^ or Fe^2+^ currents uncoupled from protons^36^, albeit small. Consistent with these observations, the ^54^Mn^2+^ assay also showed that DMT1-dependent uptake was more rapid at an acidic pH than a neutral one (Fig. 6B), consistent with DMT1’s well-established H^+^-dependence.

PGSK quenching by Fe^2+^ in mitochondria from Dox-induced cells was more sensitive to the competitive DMT1-inhibitor CISMBI (Fig. 4) at a concentration that completely inhibits Cd^2+^ uptake into hDMT1-overexpressing HEK293 cells^35^. Similarly, ^54^Mn^2+^ uptake was sensitive to sub-nanomolar levels of XEN602 (Fig. 6C), another specific DMT1 inhibitor^37^. If one assumes that 10 nM XEN602 completely inhibited DMT1 Mn transport, a rough calculation indicates that 90% of Dox-induced Mn transport is due to DMT1 and 75% of uninduced mitochondrial transport is (Fig. 6C). The residual 10% or 25%, respectively is likely to be due to an alternative XEN602 insensitive transporter. Furthermore, the second phase, but not the first phase, of PGSK quenching by Fe^2+^ displayed a half-maximal saturation at ~1.5 µM Fe^2+^ (Fig. 3), compatible with the Fe^2+^ affinity of DMT1 reported in the literature^9^. Finally, ^54^Mn^2+^ uptake was largely abolished by the Belgrade mutation in rKC mitochondria (Fig. 8). Thus there is strong evidence that DMT1 does regulate metal ion entry into mitochondria.

### Is VDAC responsible for DMT1-independent Fe^2+^ uptake?

The nature of the pronounced and fast first phase of PGSK quenching by Fe^2+^ in mitochondria (Figs 1–4, 7) is still unclear: it did not saturate up to a concentration of 25 µM, with a half-maximal effect at about 12 µM, as derived from the fit (R^2^ = 0.98) (Fig. S1). VDACs/porins have previously been proposed to mediate Fe^2+^ flux across the OMM^34^. Since VDAC is sensitive to inhibition by 4,4′-Diisothiocyano-2,2′-stilbenedisulfonic acid (DIDS)^40^, we tested the effect of this compound on Fe^2+^-induced PGSK quenching in mitochondria from both rKC and Dox-induced rDMT1-HEK293 cells. In neither preparation did DIDS (up to 1 mM) significantly affect the rapid first-phase uptake (data not shown), indicating its independence of VDAC, at least via the anion channel function that is sensitive to DIDS. Hence one can see that the mediator of phase 1 is not DMT1 nor is VDAC immediately indicted; additional pursuit of its identity is beyond the scope of this paper.

### Other implications for mitochondrial metal ion metabolism

Under conditions of pH_o_ > pH_i_, pronounced PGSK dequenching was observed during the second phase (Fig. 2A), which could be explained by several mechanisms: i. reversal of the proton-driven transport mechanism/s, ii. unmasking of a separate Fe^2+^ efflux pathway by inhibition of H^+^-coupled Fe^2+^ uptake, or iii. stimulation/activation of an Fe^2+^ export mechanism by external alkalization. Yet, possible reversibility of the direction of transport via an Slc11 family divalent metal carrier has some indirect support only for Nramp1, a protein closely related and with properties similar to DMT1^41,42^. Late second-phase PGSK dequenching observed in the presence of the DMT1 inhibitor CISMBI, however, argues in favour of a distinct mitochondrial Fe^2+^ efflux pathway. Experiments carried out in rKC mitochondria lend additional support to the existence of one or more efflux pathways for Fe^2+^ in this organelle. Thus, in this preparation, the initial influx of Fe^2+^ was followed by rapid PGSK dequenching, which was more pronounced under conditions of an outward-directed pH gradient, opposing DMT1-dependent influx (Fig. 7). Several candidates as potential mediators of mitochondrial metal export have been described in the literature. Thus, transporters possibly playing a role in iron extrusion from yeast mitochondria are Mmt1p and Mmt2p, which exhibit some homology with members of the Slc30 family of zinc transporters^43,44^. Another zinc transporter detected in mitochondria from mammalian cells and tissues is ZIP8 (Slc39A8), which accepts a number of other divalent metals besides Zn^2+^ as substrates at low micromolar concentrations, including Fe^2+^ (K_0.5_ = 0.7 µM) and Mn^2+^ ^45^. Interestingly, ZIP8 was upregulated by a post-transcriptional mechanism after cellular iron loading in H4IIE rat hepatoma cells^45^, a response that would be compatible with a role of ZIP8 in protection of mitochondria against Fe overload. Interestingly, preliminary experiments (unpublished data) indicate that Zn^2+^ and particularly Cd^2+^ uptake by isolated mitochondria from rat kidney cortex could be due to divalent metal ion transport by DMT1^7,9^, import that displays physiological and toxicological significance.

Evidence for DMT1-dependent flux of metal ions into mitochondria does not rely on the “kiss and run” hypothesis^13,46^ that proposes that endosomes and lysosomes dock with mitochondria to deliver Fe^2+^ directly, nor does the “kiss and run” hypothesis depend on DMT1. Nevertheless, it is attractive to speculate that endosomal DMT1 interacting with OMM DMT1 contributes not only to the docking but also provides a source of protons to drive the process. Recently, the group that originated “kiss and run” provided more support for the hypothesis in erythroid cells^13^. It is interesting in this context to note that mitochondrial DMT1 levels do increase in erythroid cells at a time when they are developmentally most committed to heme synthesis (Fig. S2).

Taken together, the present data provide multiple lines of evidence for DMT1 expression in the OMM and its involvement in mitochondrial Fe^2+^ and Mn^2+^ uptake in cells from various species and tissue origins (^22,23 ^and this study), while supporting the existence of additional divalent metal transport mechanisms in the OMM. Hence DMT1 function would thus be expected directly to promote mitochondrial energy production as well as antioxidant defence.

## Electronic supplementary material

Dataset 1
