# Supplementary material for: A role for divalent metal transporter (DMT1) in mitochondrial uptake of iron and manganese
Source: Sci Rep. 2018 Jan 9;8:211. doi: 10.1038/s41598-017-18584-4 (PMC5760699; doi:10.1038/s41598-017-18584-4)
Supplement: Supplementary file 1 — Dataset 1 [file 41598_2017_18584_MOESM1_ESM.doc]

**A role for divalent metal transporter (DMT1) in mitochondrial uptake of iron and manganese**

Natascha A. Wolff,^1^ Michael D. Garrick,^2*^ Lin Zhao, ^2^ Laura M. Garrick, ^2^ Andrew J. Ghio,^3^ and
Frank Thévenod ^1*^

^1^Department of Physiology, Pathophysiology & Toxicology and Center for Biomedical Education and Research (ZBAF), University of Witten/Herdecke, D-58453 Witten, Germany

^2^Department of Biochemistry, State University of New York (SUNY), Buffalo, New York 14214, USA

^3^National Health and Environmental Effects Research Laboratory, Office of Research and Development, U.S. Environmental Protection Agency, Chapel Hill, North Carolina 27599–7315, USA

**Supplementary figures**

**
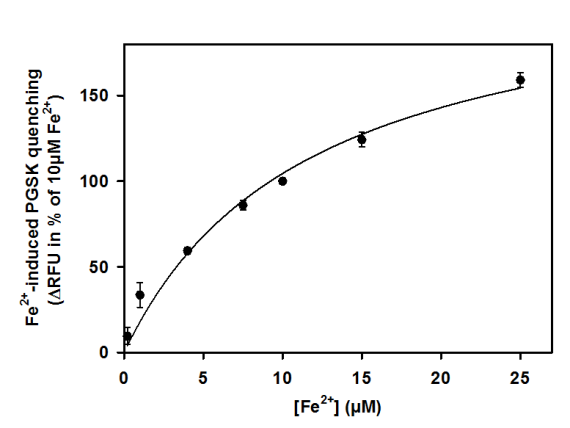
**

**Figure S1. Apparent affinity of first phase Fe^2+^ uptake in mitochondria from rDMT1-overexpressing HEK293 cells is not compatible with DMT1-mediated transport.**

Experiments were performed as described in Fig. 3. The initial 15s-slope of first-phase PGSK quenching (-ΔPGSK) was normalized to values obtained at 10µM Fe^2+^. Data are means ± SEM of 4-7 independent experiments. Data were fitted to a one-site saturation binding function, as described in Fig. 3.

**
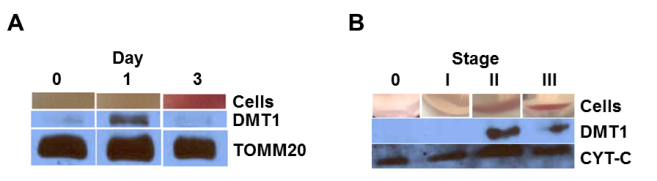
**

**Figure S2. Erythroid differentiation leads to high mitochondrial DMT1 levels early after commitment.**

**A.** MEL cells - Differentiation leads to high mitochondrial DMT1 levels before full hemoglobinization. Cells harvested just before (day 0) and after 1 and 3 days of exposure to DMSO go from very little color initially to pink (day 1) and red (day 3) during the model erythroid differentiation; whereas mitochondrial DMT1 in western blots from isolated mitochondria clearly peaks on day 1. (Days were reordered in processing – see original in Supplemental Information.) TOMM20, an OMM marker, remains constant. Apparent MW’s were ~70kDa (DMT1 detected with ex2 antibody) and ~18 kDa (TOMM20). Quantitation of **A** and a 2nd blot with a different anti-DMT1 (4ec) with day 0 DMT1/TOMM20 set as 1.0, gives day 1 = 12.2 or 13.2 and day 3 = 2.3 or 1.9 while a second experiment gave comparable results for just ex2 anti-DMT1. **B.** CD34 cells - Differentiation leads to high mitochondrial DMT1 levels before full hemoglobinization. Lee et al. ^1^ define expansion as stage 0 and use different cytokine cocktails to stimulate the cells to pass through 3 stages (I, II, III) for which one can see no hemoglobin at I, but increasing levels at II and III where the cells are virtually all erythroblasts. Mitochondrial DMT1 (MW ~67 kDa detected with ex2 antibody) peaks at stage II after being nearly invisible at 0 and I; while after stripping and restaining, cytochrome C (MW ~12 kDa) serves as a load control. Quantitation of **B** and the same blot after stripping and restaining with a different anti-DMT1 (4ec) with stage 0 DMT1/CYT-C set as 1.0, gives I = 0.16 or 0.68, II = 43 or 37 and III = 27 or 20. Absence of plasma membrane markers like the Na/K-ATPase (not shown) rules out contamination by this fraction with original images for these in Supplemental Information.

**
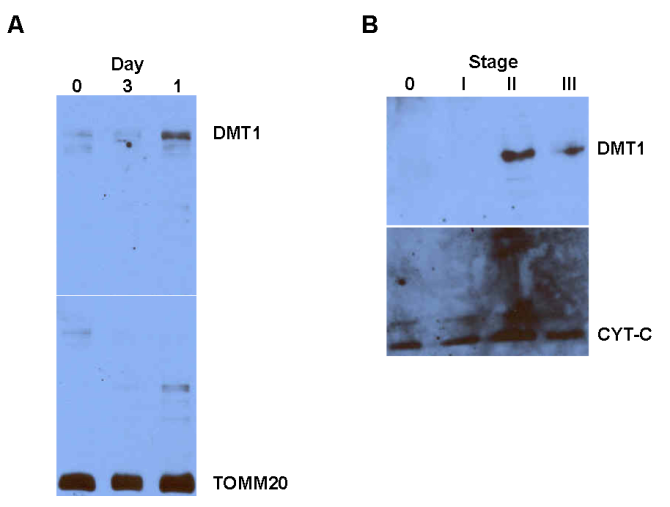
**

**Supplemental Information for Figure S2 - Erythroid differentiation leads to high mitochondrial DMT1 levels early after commitment.**

**A.** MEL cells - Differentiation leads to high mitochondrial DMT1 levels before full hemoglobinization. These are the original immunoblots for Figure S2A with the description already in the legend for that figure except to note that the order for Days 1 and 3 was unintentionally incorrect. **B.** CD34 cells - Differentiation leads to high mitochondrial DMT1 levels before full hemoglobinization. These are the original immunoblots for Figure S2B with the description already in the legend for that figure.

1 Lee, H. Y. *et al.* PPAR-alpha and glucocorticoid receptor synergize to promote erythroid progenitor self-renewal. *Nature* **522**, 474-477 (2015).
